# Clinical Comparison between HD-tDCS and tDCS for Improving Upper Limb Motor Function: A Randomized, Double-Blinded, Sham-Controlled Trial

**DOI:** 10.1155/2024/2512796

**Published:** 2024-03-31

**Authors:** Yaqin Zeng, Ruidong Cheng, Li Zhang, Shan Fang, Shaomin Zhang, Minmin Wang, Qian Lv, Yunlan Dai, Xinyi Gong, Feng Liang

**Affiliations:** ^1^Center for Rehabilitation Medicine, Rehabilitation & Sports Medicine Research Institute of Zhejiang Province, Department of Rehabilitation Medicine, Zhejiang Provincial People's Hospital (Affiliated People's Hospital), Hangzhou Medical College, Hangzhou, Zhejiang, China; ^2^Qiushi Academy for Advanced Studies, Zhejiang University, Hangzhou, Zhejiang, China

## Abstract

**Background:**

Stroke is a common and frequently occurring disease among middle-aged and elderly people, with approximately 55%−75% of patients remaining with upper limb dysfunction. How to promote the recovery of motor function at an early stage is crucial to the life of the patient.

**Objectives:**

This study aimed to investigate whether high-definition transcranial direct current stimulation (HD-tDCS) of the primary motor cortex (M1) functional area in poststroke patients in the subacute phase is more effective in improving upper limb function than conventional tDCS.

**Methods:**

This randomized, sham-controlled clinical trial included 69 patients with subcortical stroke. They were randomly divided into the HD-tDCS, anodal tDCS (a-tDCS), and sham groups. Each group received 20 sessions of stimulation. The patients were assessed using the Action Research Arm Test, Fugl–Meyer score for upper extremities, Motor Function Assessment Scale, and modified Barthel index (MBI) pretreatment and posttreatment.

**Results:**

The intragroup comparison scores improved after 4 weeks of treatment. The HD-tDCS group showed a slightly greater, but nonsignificant improvement as compared to a-tDCS group in terms of mean change observed in function of trained items. The MBI score of the HD-tDCS group was maintained up to 8 weeks of follow-up and was higher than that in the a-tDCS group.

**Conclusion:**

Both HD-tDCS and a-tDCS can improve upper limb motor function and daily activities of poststroke patients in the subacute stage. This trial is registered with ChiCTR2000031314.

## 1. Introduction

Functional impairment of the upper limbs, a common sequela after stroke, affects approximately 85% of survivors [1] and impacts their activities of daily living (ADLs) and quality of life [2]. However, restoring upper limb function, particularly the hands, is still a research focus and a rehabilitation challenge for stroke management. Transcranial direct current stimulation (tDCS), a noninvasive brain stimulation technology, regulates the excitability of the central nervous system via constant and low-intensity direct currents [3, 4]. Its clinical integration is facilitated by low costs, low safety risks, and the potential to be applied concurrently during rehabilitation [5]. The application of tDCS is simple and easy, and it is widely used in the field of neural rehabilitation [6]. Further, tDCS can promote neural plasticity by regulating the long-term potentiation depression phenomena of cortical excitability after stroke, which can modulate neuronal inhibitory and excitatory networks and has shown positive effects in improving paralytic upper limb motor function in stroke patients [7, 8], especially improvement of ADL performance [9–11]. And is more effective in treating subacute stroke than chronic stroke [12]. In contrast, high-definition transcranial direct current stimulation (HD-tDCS) has recently been developed; HD-tDCS replaces the conventional sponge electrode with small circular ones, which reduces the current coverage, results in higher spatial focusing and a longer lasting aftereffect [1, 13]. Furthermore, HD-tDCS has better stimulation focality and targeting ability [14, 15], and can improve the function of the lesioned corticospinal tract and reduce the excitability of the contralesional cortico-reticulospinal tract [16], which significantly exceeds both the magnitude and duration of conventional tDCS in healthy individuals [1], and HD-tDCS is proved safety and therapeutic potential for poststroke [17]. Conventional tDCS has been applied in stroke rehabilitation for a long time [18, 19], unlike HD-tDCS, which is not widely used in clinical practice. In patients with stroke, HD-tDCS has a positive effect on aphasia [20–22]; however, studies on HD-tDCS for the treatment of upper limb motor dysfunction following stroke are relatively rare, and the effect of treatment remains unclear. Therefore, this study aimed to explore and compare the clinical effects, costs, and safety risks between HD-tDCS and conventional tDCS in upper limb motor function after subacute stroke.

## 2. Materials and Methods

This pilot randomized controlled trial, involving three parallel groups, was performed in accordance with the CONSORT guidelines for randomized pilot and feasibility trials, and was conducted in Zhejiang Provincial People's Hospital (China) from March 2019 to December 2021. No changes to the methods occurred after the pilot trial commenced.

### 2.1. Participants

Patients with stroke who were hospitalized in the Department of Rehabilitation Medicine at Zhejiang Provincial People's Hospital from 2019 to 2021 were included in this study. All patients met the diagnostic criteria for stroke as defined at the Fourth National Conference on Cerebrovascular Diseases [23]. The inclusion criteria for this study were as follows: (i) patients with cerebral hemorrhage and cerebral infarction, as confirmed by computed tomography or magnetic resonance imaging (MRI); (ii) patients aged 18–70 years with a disease course ranging from 2 weeks to 3 months; and (iii) patients with unilateral hemiplegia, with a grade of ≤I for the affected side on the modified Ashworth Scale of muscle tension. Exclusion criteria for this study were as follows: (i) unstable vital signs; (ii) use of implantable electronic devices, such as cardiac pacemakers; (iii) patients with metal implants, such as stents; (iv) patients with critical illness or major organ failure; (v) pregnant women; (vi) patients with severe allergy to electrode patch, local skin injury or inflammation, and hyperalgesia in the stimulated area; (vii) patients with epilepsy or secondary epilepsy with definite diagnosis; (viii) patients with tumors; (ix) patients with severe cognitive impairment; and (x) patients with a history of mental disorders or using medications that could cause symptoms of mental disorders. Consumption of drugs that could affect the central nervous system was prohibited during the study period.

### 2.2. Research Design

This study was a double-blinded, randomized controlled trial that explored the therapeutic effects of HD-tDCS and anodal tDCS (a-tDCS) on upper limb dysfunction after stroke. Patients were randomly assigned to the HD-tDCS, a-tDCS, and sham groups using computerized random blocks. An independent researcher, who was not involved in the tDCS administration or outcome assessments, performed the randomization by applying a number sequence. The participants and investigators were blinded to the treatment allocation.

### 2.3. Intervention Measures

All patients received routine rehabilitation, including 30-min acupuncture, 20-min physical factor therapy, 40-min exercise therapy, and 40-min occupational therapy.

### 2.4. HD-tDCS Group

This group of patients was treated with the 4 × 1 high-definition transcranial electrical current stimulator from Soterix Medical (New York, NY). Its central electrode is the stimulation electrode, and the other four are the receiving electrodes. Before treatment, transcranial magnetic stimulation (TMS; CCY-I TMS instrument, Yiruide Co., Ltd., Wuhan, China) was used to stimulate the primary motor cortex (M1) area on the damaged side with a single pulse, measure the motor evoked potential (MEP) of the abductor pollicis brevis, and determine the cortical stimulation hot spot; when the MEP could not be detected, the anode was placed in the C3/C4 area of the motor cortex [24]. According to the electroencephalogram (EEG) 10–20 system, the intersection of the connecting line from the nasal root to the occipital tuberosity and that between the anterior points of the left and right ear was defined as CZ. Patients wore EEG caps during this study. The anode was placed on the stimulation point determined by TMS (point C3 or C4 in the motor area). The four receiving electrodes were placed at C1/C2, C5/C6, FC3/FC4, and CP3/CP4, approximately 3.5 cm in the front, back, left, and right of the motor area. The stimulating electrode can transmit 1.5 mA current, and the four receiving electrodes can transmit 0.4, 0.4, 0.4, and 0.3 mA, respectively [25, 26]. The duration between the uphill current before the commencement of stimulation and the downhill current following stimulation to reduce the patient's discomfort was 30 s.

### 2.5. a-tDCS Group

A 1 × 1 DC stimulator from Soterix Medical (New York, NY) was used to perform a-tDCS using two 5 cm × 7 cm isotonic saline gelatin sponges with two 5 cm × 5 cm rubber electrodes. Following a stroke, the anode was placed on the damaged hemisphere (C3 or C4 point of motor area) and the cathode on the contralateral orbit [27].

### 2.6. Sham Group

**Figure 1:**
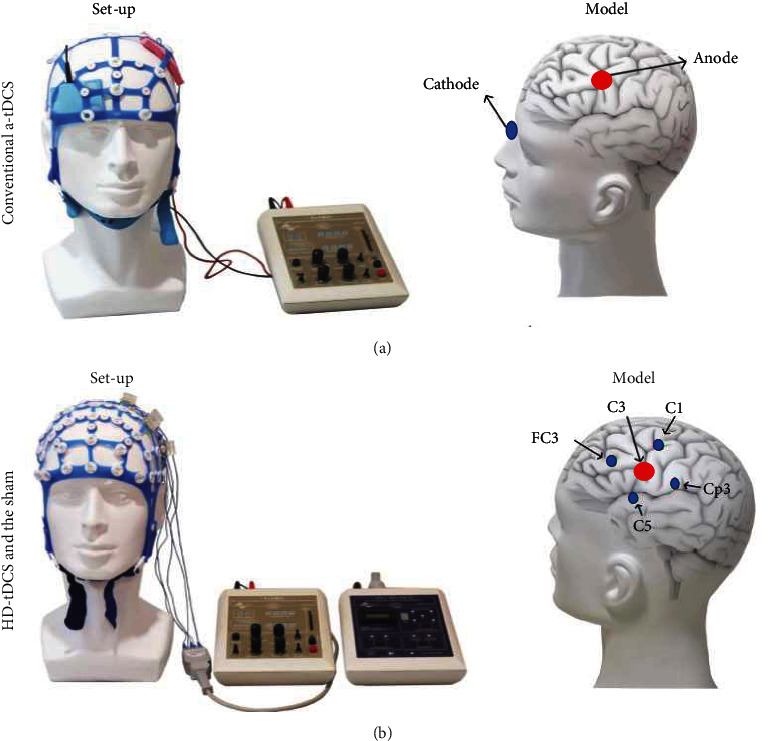
Treatment of patients with HD-tDCS, a-tDCS, and sham stimulation. Red denotes anodal stimulation, whereas blue denotes cathodal stimulation. (a) Conventional a-tDCS was performed using two 5 cm × 7 cm isotonic saline gelatin sponges with two 5 cm × 5 cm rubber electrodes in a 10–20 EEG cap and (b) HD-tDCS was performed using EEG-sized electrodes held in plastic insets in a 10–20 EEG cap. A 4 × 1 channel HD-tDCS prototype device was used in the study. EEG, electroencephalogram; HD-tDCS, high-definition transcranial direct current stimulation; and a-tDCS, anodal transcranial direct current stimulation.

In this group, the stimulator was automatically interrupted after outputting 1.5 mA current for 15 s, while the other steps were the same as those for HD-tDCS (Figure 1).

### 2.7. Blinding Procedure

A double-blinded design was used in the study, wherein the patients were blinded to the type of stimulation they received. A rater who was blinded to the stimulation performed evaluations at three separate intervals: before, after 4 weeks of treatment, and at the 8-week follow-up. The stimulation times for these three groups were 20 min, once a day, five times a week, and the treatment lasted for 4 weeks, resulting in a total of 20 treatment sessions.

### 2.8. Assessment

The patients were evaluated within 72 hr before and after treatment. The same trained physician, blinded to patient groupings, conducted all evaluations. The Action Research Arm Test (ARAT) [28] is a commonly used evaluation method for fine hand motor function [29]. It focuses more on assessing the fine hand function and is often used as a secondary outcome indicator in combination with the Fugl–Meyer score for the upper extremity (UEFM) [30]. The scale is divided into four parts: grasp, grip, pinch, and gross, totaling 19 items. The total score was set to 57, and a higher score indicated better upper limb motor function recovery.

The UEFM is the most commonly used outcome index for evaluating the stroke-associated function of the upper limb. The scale includes reflex, sensation, and motor function [31], comprising 33 items in total. The total score was set to 66 points, and a lower score indicated more severe upper limb motor dysfunction. The Motor Function Assessment Scale (MAS) [32, 33] consists of eight different motor function items, one of which is related to systemic muscle tension. General muscle tension items are not included in the total score and are only used for reference. Except for general muscle tension, each item is scored 0–6 points, with a total score of 48 points. The higher the score, the better the motor function. As a tool, the modified Barthel index (MBI) has good reliability and validity in evaluating the ADL of patients with stroke [34]. It includes 10 items: feeding, dressing, grooming, toilet use, bowel control, bladder control, transfer (bed to chair and back), activity, going up and down stairs, and bathing. According to the patient's care and dependance, the score is 0/5/10/15, and the total score ranged from 0 to 100, with a higher score indicating a higher functional ability.

### 2.9. Statistical Analysis

Statistical analyses were performed using SPSS version 26 (IBM Corp., Armonk, NY, USA). Demographic characteristics and baseline data were compared among groups using the *χ*^2^ test, one-way analysis of variance (ANOVA), and Kruskal–Wallis test. Before and after treatment, the paired sample *t*-test was used for intragroup comparison for normally distributed data, and Wilcoxon signed rank test was conducted as a nonparametric equivalent test. For comparison among groups, a one-way ANOVA was used for normally distributed data, whereas Kruskal–Wallis test was used as a nonparametric equivalent test. All data were quantitatively interpreted as median, quartile, mean, and standard deviation. A *P* value of 0.05 or less indicated statistical significance. Finally, an a priori analysis was performed to obtain the estimated sample size to inform future tDCS clinical trials. We used the *α* level = 0.05 and *β* = 0.5 to calculate the differential amount of change among the HD-tDCS, a-tDCS, and sham groups on the UEFM scale to measure the primary outcomes of clinical efficacy. All analyses were performed using SPSS (Version 26) and *G* ^*∗*^ Power 3. 1 software with significance level set at *P*  < 0.05.

## 3. Results

**Figure 2:**
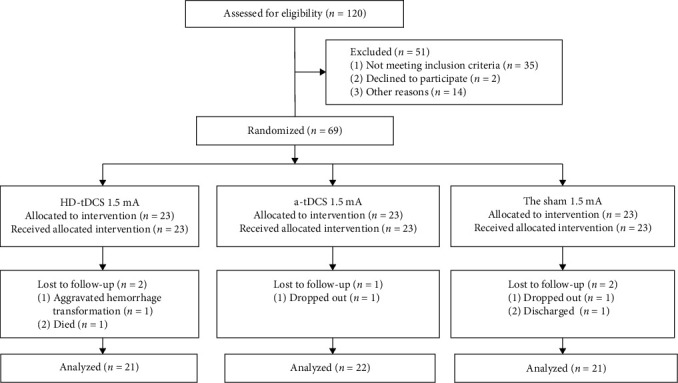
Flow diagram illustrating the study design. No significant differences in sex, age, disease course, stroke hemisphere, stroke type, Brunnstrom stage, and other general data were observed among the three groups (Table 1). The ARAT, UEFM, MAS, and MBI scores of the three groups were determined before and after 4 weeks of stimulation, and the patients' MBI scores were analyzed after 8 weeks of follow-up. One-way ANOVA or the Kruskal–Wallis test was conducted to analyze the recovery of patients in the three groups during treatment. On intragroup comparison, the scores showed significant differences after 4 weeks of treatment compared with those before treatment (*P* < 0.05). ARAT, action research arm test; UEFM, Fugl–Meyer score for the upper extremity; MAS, Motor Function Assessment Scale; MBI, modified Barthel index; and ANOVA, analysis of variance.

Of the 120 patients with stroke, 69 were selected and randomly divided into the HD-tDCS, a-tDCS, and sham groups, with 23 patients per group. However, only the data of 64 patients were finally analyzed (Figure 2) following five cases of withdrawal and loss of follow-up. No serious adverse reactions related to the program were reported. For this study, the primary outcome was improvement in upper limb movement and ADL, as assessed using the ARAT, UEFM, and MBI. The secondary outcome was the improvement evaluated using the MAS. No significant differences in sex, age, disease course, stroke hemisphere, stroke type, Brunnstrom stage, and other general data were observed among the three groups (Table 1). The ARAT, UEFM, MAS, and MBI scores of the three groups were determined before and after 4 weeks of stimulation, and the patients' MBI scores were analyzed after the 8-week follow-up. One-way ANOVA or Kruskal–Wallis test was conducted to analyze the recovery of patients in the three groups during the treatment. On intragroup comparison, the scores showed significant difference after 4 weeks of treatment, as compared with those before treatment (*P*  < 0.05).

**Figure 3:**
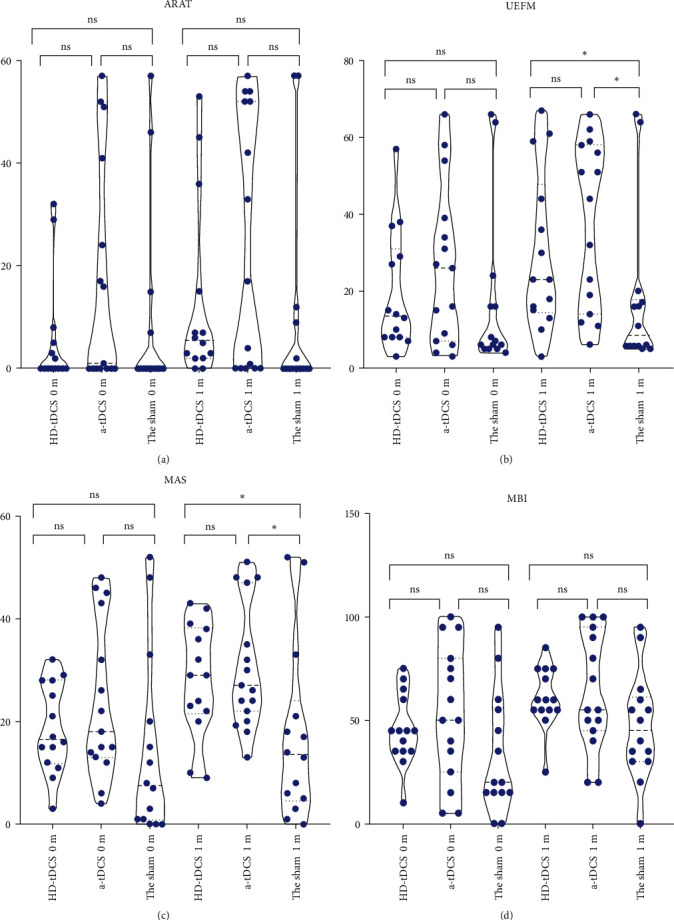
Clinical scales for patients at different time points. The UEFM and MAS scores improved after HD-tDCS or a-tDCS treatment compared with those after sham treatment.  ^*∗*^*r* < 0.05. HD-tDCS, high-definition transcranial direct current stimulation; a-tDCS, anodal transcranial direct current stimulation; UEFM, Fugl–Meyer score for the upper extremity; MAS, motor function assessment scale; MBI, modified Barthel index; ARAT, action research arm test; and ns, not significant.

The therapeutic effects in the HD-tDCS and a-tDCS groups were similar (Table 2 and Figure 3). In addition, the UEFM (*P*=0.028), MAS (*P*=0.032), and MBI (*P*=0.018) scores after treatment were significantly higher in the HD-tDCS group than in the sham group. The UEFM (*P*=0.035) and MAS (*P*=0.031) scores were significantly higher in the a-tDCS group than in the sham group. However, the ARAT (*P*=0.864), UEFM (*P*=0.903), MAS (*P*=0.99), and MBI (*P*=0.372) scores were not significantly different between the HD-tDCS and a-tDCS groups. After 8 weeks of follow-up, the MBI scores in the HD-tDCS group were higher than those in the a-tDCS and sham groups (*P*=0.014).

## 4. Discussion

In this study, we showed that HD-tDCS or a-tDCS-based noninvasive electrical stimulation has advantages over the conventional treatment in improving the upper limb motor function of patients with stroke. HD-tDCS is a new electrotherapy method regarded as the first noninvasive and targeted neuromodulation technology [14]. With advancements in medical technology, HD-tDCS has been gradually employed in the clinic. Recent research results have shown that using multiple small electrode sheets can improve the spatial resolution of tDCS [33, 34], optimize the action position and intensity of the applied current, obtain adequate and targeted stimulation, and ensure stimulation safety [35].

tDCS, a noninvasive and low-intensity brain stimulation technology, involves using 1–2 mA constant microcurrent to regulate the activity of cerebral cortical nerve cells. It can stimulate the cerebral cortex through the anode, change the potential difference inside and outside the neuron membrane, promote the excitation and discharge of nerve cells, and regulate the activity of the cerebral cortex. Additionally, it improves motor function after stroke [36–38]. Further, we found that HD-tDCS, similar to conventional tDCS, could improve upper limb motor function and ADLs of poststroke patients in the subacute phase, thus providing a new rehabilitation therapy for limb dysfunction. Nevertheless, the neural mechanisms by which tDCS improves limb activity remain unclear but may include the following [39–44]:

1. Changes in neuronal membrane potential: anodic stimulation can depolarize neuronal membrane potential and increase cortical excitability. In contrast, cathodic stimulation can hyperpolarize neuronal membrane potential and reduce cortical excitability [43].
2. Neurotransmitter mechanism: tDCS can reduce the concentration of *γ*-aminobutyric acid in the cerebral cortex, thereby decreasing the inhibition ability of the cortical loop, changing the excitability in the cortex, and improving the excitability of the cortical loop [44]. The results showed that *γ*-aminobutyric acid level negatively correlates with functional connections' strength in resting motor networks [45].
3. Regulating brain plasticity by mediating the subthreshold of neuronal resting membrane potential: tDCS induces the glutamate receptor N-methyl-D-aspartate and *α*-amino-3-hydroxy-5-methyl-4-isoxazole-propionic acid functions and undergoes polarity dependent modification, thereby increasing glutamate sensitivity and regulating synaptic plasticity [46].
4. Increased cerebral blood flow: tDCS can change local cerebral blood flow, increase cerebral blood flow perfusion acting on the corresponding areas of the dorsolateral prefrontal cortex, improve cerebral blood supply by regulating neurovascular units or regulating cerebral microvessels, and enhance the functional connection of the premotor, motor, and sensory-motor areas of its hemisphere.

The results of the ARAT evaluation indicated no statistical difference among the three groups before and after the intervention; this may be because the onset time after stroke was only 40 days in the patients included in this study. At the onset of this study, part of Brunnstrom's hand function in the enrolled patients was at stage II, indicating that the patients only had very slight flexion and extension hand functions. Owing to the complex requirements for ARAT evaluation of hand function and the poor hand motor function at baseline, item evaluation has become more challenging, which may explain the absence of statistical differences among the groups in the ARAT evaluation of hand flexibility [47]. In the sham group, the MAS and MBI scores significantly increased, which may be because more attention was paid to the training and improvement of lower limb function, such as walking and transferring, in the clinical rehabilitation treatment for patients in the subacute stage following stroke [48]. Additionally, the UEFM scores of the HD-tDCS and a-tDCS groups were higher after treatment than before treatment and were better than those of the sham group, indicating that the neuromodulation technology could effectively restore the motor function of the upper limbs and hands in the early stage of subacute stroke.

After 4 weeks of treatment, improvement in upper limb function for the HD-tDCS group was equivalent to that of the a-tDCS group, which may be because of the following reasons. First, the change in current was primarily caused by the individual morphological differences between the cerebrospinal fluid and the brain. It is impossible to achieve a unified standard in the experiment as it is necessary to locate the current according to the detailed image; otherwise, it will be impossible to detect the differences between these two groups as they are affected by the different anatomies of patients [49]. Second, the study compared a-tDCS and HD-tDCS according to the finite element model analysis (FEM) based on high-resolution MRI [50], which may have influenced the observed results. Although HD-tDCS can be used to target cortical motor areas, which could significantly improve focusing and specificity, the stimulation depth was not as deep as that in conventional a-tDCS [13]. In addition, there were differences between the electrode or conductive paste and the scalp during treatment, which may have affected the results to a certain extent [51]. Third, the time of excitatory changes between a-tDCS and HD-tDCS treatments was different. Cortical spinal cord excitability peaked immediately after conventional tDCS treatment and gradually returned to baseline [1, 52]. HD-tDCS-induced plasticity peaked at approximately 30 min after stimulation, and its aftereffect duration was longer than that of conventional tDCS. Therefore, the observed disparity between the results for the a-tDCS and HD-tDCS groups may be attributable to the different results in the time sequences postintervention evaluation.

Compared with those in the a-tDCS and sham tDCS groups, the MBI scores significantly increased after 8 weeks of follow-up in the HD-tDCS group (*r* = 0.02). The results revealed that HD-tDCS was effective in improving upper limb movement and ADL, with a sustained effect at 4-week and 8-week follow-ups, suggesting that HD-tDCS may lead to longer-lasting TMS-evoked MEP changes and may have a longer aftereffect duration than a-tDCS [1]. Additionally, preliminary efficacy data indicated that HD-tDCS effects were at least comparable to those of a-tDCS, and the cost of consumables was equivalent. However, compared with a-tDCS, HD-tDCS had longer-lasting stimulation and more significant excitability, considerably reducing overall perceived pain among patients with stroke, which is more acceptable. Therefore, we believe that HD-tDCS is a promising clinical technique and warrants further investigation.

The results obtained using various clinical scales, such as ARAT, UEFM, MAS, and MBI, indicated that HD-tDCS and a-tDCS exert comparable therapeutic effects in improving upper limb motor function in poststroke patients. However, these clinical scales may not be sufficiently sensitive to show the effects of HD-tDCS and a-tDCS (especially HD-tDCS with respect to focal motors). In the future, objective indicators, including electromyography and kinematic measurements, could be applied to evaluate the activation change in M1 before and after tDCS treatment, and to reveal the mechanism underlying the difference in effects. Neurophysiological factors could be another possible reason for the lack of difference between the two tDCS approaches. The improvement in motor performance is often accompanied by neuroplastic adaptations in the central nervous system, and tDCS induces neuroplasticity by modulating the activity of brain structures [53]. Our results indicated that the MBI scores of the HD-tDCS group at 8 weeks of follow-up were better than those of the a-tDCS group, suggesting that HD-tDCS enhanced the process of neuroplasticity better than a-tDCS. Neuroplasticity may be sufficient to improve motor function, and this process may require more time than 4 weeks to show differences in ARAT, UEFM, and MAS scores. This may be why no significant differences in ARAT, UEFM, MAS, and MBI scores were observed between the HD-tDCS and a-tDCS groups after 4 weeks. In the future, task-functional MRI could be used to delineate the differences in M1 excitability determined by the stimulation.

Our study had some limitations. As we only followed up on MBI, incomplete follow-up would affect the final results of patients. Additionally, heterogeneity could not be excluded for cases collected only in one center. The clinical scales were not sufficiently specific to investigate how focal motors improve, and they lacked sensitivity to show the effects, which would affect the final results.

## 5. Conclusions

HD-tDCS and a-tDCS showed comparable therapeutic effects for improving upper limb motor function in poststroke patients. Additionally, improvements in MBI were more prominent and longer-lasting in participants receiving HD-tDCS than in those receiving a-tDCS or sham, thus providing a new therapeutic method for improving upper limb function in poststroke patients.

## Figures and Tables

**Table 1:** Clinical data and preliminary evaluation of three patient groups.

		Mean (SD) or median (IQR)		
	HD-tDCS (25%, 75%)	a-tDCS (25%, 75%)	Sham (25%, 75%)	*r*
*N*	21	22	21	—
Age (years)	55.14 ± 14.76	57.73 ± 12.14	57.24 ± 13.75	0.805^b^
Sex, male/female	8/13	14/8	15/6	0.072^a^
Hemisphere, left/right	6/15	13/9	7/14	0.089^a^
Type, ischemic/hemorrhage	11/10	16/6	11/10	0.29^a^
Stroke interval (weeks)	1.25 (1, 2.25)	1.25 (1, 2)	2 (1, 2.25)	0.599^c^
Brunnstrom stage				
Hand	2 (1.5, 3)	2.5 (1, 4)	2 (1, 3)	0.603^c^
Upper limb	2 (1, 3)	2 (1, 3.25)	2 (1, 3)	0.943^c^
ARAT	0 (0, 6.5)	0 (0, 18.75)	0 (0, 11)	0.8^c^
UEFM	16 (11.5, 30.5)	15.5 (7, 34.25)	8 (6, 25)	0.115^c^
MAS	20 (12.5, 26.5)	15 (13, 27.5)	12 (5, 21)	0.179^c^
MBI	40.71 ± 20.27	42.5 ± 27.11	33.1 ± 23.64	0.4^b^

ARAT, action research arm test; UEFM, Fugl–Meyer score for the upper extremity; MAS, motor function assessment scale; MBI, modified Barthel index; SD, standard deviation; HD-tDCS, high-definition transcranial direct current stimulation; and a-tDCS, anodal transcranial direct current stimulation. ^a^*Chi*-square test. ^b^One-way ANOVA; *P* < 0.05. ^c^Kruskal–Wallis test; *P* < 0.05.

**Table 2:** Time-related variables of all three groups (Mean (SD) or median (IQR)).

	HD-tDCS (25%, 75%)	a-tDCS (25%, 75%)	Sham (25%, 75%)	*r*
ARAT				
* T*_onset_	0 (0, 6.5)	0 (0, 18.75)	0 (0, 11)	0.8
* T*_end_	7 (2, 27)	4.5 (0, 45.75)	0 (0, 14.5)	0.259
UEFM				
* T*_onset_	16 (11.5, 30.5)	15.5 (7, 25)	8 (6, 25)	0.115
* T*_end_	30 (16.5, 53.5)	27.5 (14.75, 53.75)	16 (6, 23)	0.045
MAS				
* T*_onset_	20 (12.5, 26.5)	15 (13, 27.5)	12 (5, 21)	0.179
* T*_end_	29.05 ± 10.1	29 ± 10.44	20.71 ± 15.68	0.047
MBI				
* T*_onset_	40.71 ± 20.27	42.5 ± 27.11	33.10 ± 23.64	0.4
* T*_end_	63.1 ± 17.5	55 ± 23.2	49.29 ± 21.35	0.106
* T*_follow_	78.57 ± 16.06	66.14 ± 21.27	60.95 ± 20.16	0.014

ARAT, action research arm test; UEFM, Fugl–Meyer score for the upper extremity; MAS, motor function assessment scale; MBI, modified Barthel index; SD, standard deviation; HD-tDCS, high-definition transcranial direct current stimulation; and a-tDCS, anodal transcranial direct current stimulation.

## Data Availability

The full data set generated or analyzed in this study can be obtained by contacting the corresponding author Feng Liang.
